# Peroxisomal-dependent signalling and dynamics modulate plant stress responses: reactive oxygen and nitrogen species as key molecules

**DOI:** 10.1093/jxb/eraf072

**Published:** 2025-02-24

**Authors:** Eliana Molina-Moya, Alejandro Rodríguez-González, María A Peláez-Vico, Luisa M Sandalio, María C Romero-Puertas

**Affiliations:** Department of Stress, Development and Signalling in Plants, Estación Experimental del Zaidín (EEZ), Consejo Superior de Investigaciones Científicas (CSIC), C/Prof. Albareda, 1, 18008, Granada, Spain; Department of Stress, Development and Signalling in Plants, Estación Experimental del Zaidín (EEZ), Consejo Superior de Investigaciones Científicas (CSIC), C/Prof. Albareda, 1, 18008, Granada, Spain; Division of Plant Sciences and Technology, College of Agriculture Food and Natural Resources and Interdisciplinary Plant Group, University of Missouri, Columbia, MO 65211, USA; Department of Stress, Development and Signalling in Plants, Estación Experimental del Zaidín (EEZ), Consejo Superior de Investigaciones Científicas (CSIC), C/Prof. Albareda, 1, 18008, Granada, Spain; Department of Stress, Development and Signalling in Plants, Estación Experimental del Zaidín (EEZ), Consejo Superior de Investigaciones Científicas (CSIC), C/Prof. Albareda, 1, 18008, Granada, Spain; Université Côte d'Azur, France

**Keywords:** Catalase, dynamics, hydrogen peroxide, nitric oxide, peroxisomes, reactive nitrogen species, reactive oxygen species

## Abstract

Plant peroxisomes are organelles housing different key metabolic pathways in the cell such as photorespiration and fatty acid β-oxidation. The metabolism of phytohormones, polyamines, and other key signalling molecules such as reactive oxygen and nitrogen species (ROS and RNS) takes place in these organelles. The presence of a complex antioxidant system that may regulate the ROS/RNS level makes peroxisomes key organelles governing ROS/RNS-dependent signalling. The evolution of -omics technologies and the existence of mutants with specifically altered ROS metabolism in peroxisomes have given us a large amount of data and genes that could be regulated in plant responses to stress. All these data point to the existence of a specific transcriptomic footprint associated with peroxisomes. Furthermore, advances in microscopy and the implementation of new molecules have allowed us to visualize organelles *in vivo* and obtain detailed information about the dynamics of these organelles involving changes in their velocity, peroxule formation, and proliferation. In this review, we update the latest information about peroxisomal metabolism and signalling, mainly related to ROS/RNS under control and stress conditions and how the different stimuli affect the plasticity and dynamics of the organelles, which can contribute in turn to plant responses to these stimuli.

## Teaser

Peroxisomes are one of the principal organelles in the cell. We update information about peroxisomal metabolism, dynamics, and signalling, as related mainly to reactive oxygen and nitrogen species.

## Introduction

Observed for the first time in mouse renal cells by Rhodin in 1954 and initially called microbodies, peroxisomes were one of the last principal organelles in cells to be discovered (Rhodin, 1954). In fact, it was previously believed that peroxisomal enzymes belonged to the mitochondria (De Duve and Baudhuin, 1966). Peroxisomes originated in primitive respiratory systems adapting to an emerging oxygenated atmosphere as a result of photosynthesis. They have persisted throughout evolution as we can find them in all eukaryotes except in the Archaezoa, and they are important connectors between oxidative and biosynthetic pathways that occur in different compartments in the cell (Wayne, 2010). The peroxisome concept appeared following enzyme distribution studies, given that the first role assigned to these organelles was the capacity for detoxification of reactive oxygen species (ROS) (Gabaldón, 2018). The term ROS is coined for a group of molecules derived from oxygen reduction that occur as a normal consequence of aerobic life, and includes free radicals, such as superoxide (O_2_·^−^) or hydroxyl (·OH), and non-radicals such as hydrogen peroxide (H_2_O_2_) or singlet oxygen (^1^O_2_), among others (Halliwell and Gutteridge, 2015). More recent is the term reactive nitrogen species (RNS), in whose metabolism peroxisomes are also involved. RNS include a group of molecules derived from the reduction or oxidation of nitrogen compounds, including nitric oxide (NO), *S*-nitrosoglutathione (GSNO), and peroxynitrite (ONOO^–^) as principal molecules (Delledonne, 2005).

Depending on the cell or tissue type and the growth and developmental stage, plant peroxisomes can be classified into five categories, namely (i) glyoxysomes, associated with fatty acid (FA) β-oxidation and the glyoxylate cycle that produce sugars from stored lipids during post-germination seedling growth; (ii) leaf peroxisomes catalysing essential reactions of photorespiration and being important in photomorphogenesis; (iii) gerontosomes, located in senescent tissues that use glyoxysomal enzymes to catabolise lipids; (iv) root nodule peroxisomes, participating in nitrogen fixation in legumes by ureide biosynthesis; and (v) ‘non-specialized’ peroxisomes, which are relatively undifferentiated peroxisomes distributed throughout the entire plant (Olsen, 1998). This peroxisomal specialization observed in plants may indicate that plants actually have multiple and more complex layers of regulation compared with those observed in yeast and mammals. Currently, they are known to be key players in many different physiological processes in cell metabolism and in the responses to stress in both animal and plant organisms, which involves interorganelle communication (Sandalio and Romero-Puertas, 2015; Fransen and Lismont, 2019). Such is its importance that severe deficiency in peroxisome function or biogenesis leads to fatal human disorders and plant embryonic lethality (Wanders *et al.*, 2023). In this review, we update knowledge about peroxisome metabolism mainly focused on hormone biosynthesis and ROS and RNS metabolism, and the peroxisomal-dependent signalling in the plant responses to stress. Additionally, we will discuss how peroxisomes can perceive stress factors and the involvement of changes in their dynamics in the cell responses to stress.

## Biological and metabolic functions of peroxisomes

Since the findings by Lazarow and De Duve (1976) indicated a confusing role for peroxisomes as an auxiliary system to mitochondria, several studies have been carried out to elucidate the unique role of peroxisomes in metabolism. FA β-oxidation and H_2_O_2_ detoxification are peroxisomal features shared across kingdoms (Smith and Aitchison, 2013). Despite this, a wide range of functions have been conferred on peroxisomes, varying significantly depending on the organism and even between organs from the same organism. For example, some peroxisomal functions in yeasts consist of the oxidation of methanol (van Dijkan *et al.*, 1982) and the metabolism of primary amines (Zwart *et al.*, 1983). In filamentous fungi, some species including *Penicillium chrysogenum* contain in their peroxisomes the enzymes that produce penicillin (Müller *et al.*, 1992). Other species such as *Neurospora crassa* have specialized peroxisomes called ‘Woronin bodies’, functioning as a plug to stop leakage of the cytosol (Jedd and Chua, 2000). In mammals, key enzymes responsible for cholesterol, bile acid, and plasmalogen synthesis are found in peroxisomes (Brown and Baker, 2008). Furthermore, different roles in non-metabolic processes including ageing, antiviral defence, and cancer have also been linked to peroxisomes in humans (Kim, 2020).

Regarding peroxisomes in plants, their principal functions are connected to different metabolic pathways, such as lipid metabolism, photorespiration, H_2_O_2_ metabolism, biosynthesis of plant hormones, and assimilation of symbiotically induced nitrogen, among others (Mano and Nishimura, 2005). These organelles participate in different cellular processes involved in plant development, morphogenesis, and responses to stress (Sandalio and Romero-Puertas, 2015). For instance, the importance of plant peroxisomes in reproduction, seed development, seed germination, and early seedling establishment is related to FA β-oxidation and the production of (+)-7-iso-jasmonic acid (JA) and other signals, as has been shown in different plant species such as *Arabidopsis thaliana*, tomato, rice, maize, lily, moss, and petunia (Pan *et al.*, 2019). Furthermore, recent -omics analyses have elucidated new metabolic pathways associated with peroxisomes such as biosynthesis of biotin, phylloquinone, ubiquinone, isoprenoids, and benzoic acid (BA) derivatives, and sulfite metabolism, adding more complexity to the role of peroxisomes in metabolism (Kao *et al.*, 2018; Pan *et al.*, 2020).

## Peroxisomes as a source and modulator of plant signalling molecules

### Reactive oxygen species

Peroxisomes are an important source of H_2_O_2_, O_2_·^−^, and ·OH (Sandalio *et al*., 2021). Reactions that occur in peroxisomes during photorespiration are responsible for 70% of total H_2_O_2_ production in the cell, with the most remarkable enzymes involved being photorespiratory glycolate oxidase (GOX), present in green tissues (Foyer and Noctor, 2020); the main enzyme of FA β-oxidation, acyl-CoA oxidase (ACX); and the FAD- and FMN-dependent oxidases. Another H_2_O_2_ source in peroxisomes is spontaneous or enzymatic dismutation of O_2_·^−^, through superoxide dismutases (SODs). H_2_O_2_ is also produced in peroxisomes as a result of polyamine (PA) catabolism, as described later (Wang *et al*., 2019). Sarcosine oxidase (SOX) located in peroxisomes also contributes to H_2_O_2_ production in the organelle, involving the oxidation of sarcosine to glycine, formaldehyde, and H_2_O_2_ (Goyer *et al.*, 2004). Regarding O_2_·^−^ generation, important pathways are ureide metabolism and nucleic acid catabolism, with the implication of enzymes such as xanthine oxidoreductase (XOR) and urate oxidase (UO, also known as uricase). Other O_2_·^−^ sources are sulfite oxidation by sulfite oxidase and a small electron transport chain in the peroxisomal membrane (Sandalio and del Río, 1988; Khan *et al.*, 2023). ·OH is generated in peroxisomes as a consequence of Fenton-type reactions, and ^1^O_2_ production has also been reported in this organelle although the mechanism remains unknown (Mor *et al.*, 2014).

Plant peroxisomes have an extensive antioxidant system that consists of enzymatic and non-enzymatic components (Sandalio *et al.*, 2021). The principal enzymatic antioxidants in peroxisomes are constituted by SODs, catalase (CAT), and enzymes in the ascorbate–glutathione (ASC–GSH) cycle such as ascorbate peroxidase (APX), monodehydroascorbate reductase (MDHAR), dehydroascorbate reductase (DHAR), and glutathione reductase (GR; Jimenez *et al.*, 1997). SOD may decrease oxidative damage by catalysing the rapid dismutation of O_2_·^−^ to O_2_ and H_2_O_2_, and different metalloenzymes have been described in peroxisomes depending on the species. In particular, Arabidopsis possesses seven SOD isoenzymes, with CuZn-SOD3 (CSD3) being located in peroxisomes. The peroxisomal ASC–GSH cycle, which, in Arabidopsis, is composed of two genes coding for APXs, *APX3* and *APX5*, and one gene each coding for MDHAR (*MDHAR1*), DHAR (*DHAR1*), and GR (*GR1*; Mhamdi *et al.*, 2012; Sandalio and Romero-Puertas, 2015; Pan *et al.*, 2020), contributes to regulate H_2_O_2_ levels in the organelle. The ASC–GSH cycle also involves regulation of the non-enzymatic redox buffers GSH/GSSG and ASC/DHA in the peroxisome. CAT is mainly a peroxisomal enzyme and catalyses dismutation of H_2_O_2_ molecules into O_2_ and H_2_O. In Arabidopsis, three *CAT* genes (*CAT1*, *CAT2*, and *CAT3*) have been identified. Glutathione *S*-transferases (GSTs) also contribute to peroxide regulation in the organelles (Pan *et al.*, 2020), with three GSTs being identified (GST1–GST3) in these organelles in Arabidopsis by Strep-tagged recombinant proteins (Dixon *et al.*, 2009). No thioredoxins and/or peroxiredoxins has been located in plant peroxisomes so far, although two orthologues of human peroxisomal peroxiredoxin 5 (PRDX5) have been identified recently as genes regulated in peroxisomes affected in H_2_O_2_ metabolism (Terrón-Camero *et al.*, 2022). However, whether these peroxiredoxins are located in Arabidopsis peroxisomes needs further investigation.

### Reactive nitrogen species

Concerning RNS production in peroxisomes, an arginine-dependent NO production has been shown (Barroso *et al*., 1999). In addition, NO has been shown to be produced during indole-3-butyric acid (IBA) to indole-3-acetic acid (IAA) conversion by β-oxidation (Schlicht *et al.*, 2013). Furthermore, NO might be produced after nitrite reduction by XOR, from PA and amine oxidase activities (Wimalasekera *et al.*, 2011) and from molecules such as oximes by the activity of peroxidases (PODs) and flavins (Sandalio *et al*., 2021; López-Gómez *et al.*, 2024). NO can react with O_2_·^−^ producing ONOO^–^, as well as with GSH leading to GSNO production, which is an important cellular reservoir of NO. Both RNS (ONOO^–^ and GSNO) have been described in peroxisomes (Ortega-Galisteo *et al.*, 2012; Corpas and Barroso, 2014; Sandalio *et al.*, 2021). Urate, which has been located in peroxisomes, may contribute to regulate ONOO^–^ levels, and *S*-nitrosoglutathione reductase (GSNOR), which regulates *S*-nitrosothiol levels, has also been identified in plant peroxisomes by proteomic analysis (reviewed in Sandalio *et al.*, 2019). Different reports provided evidence about the regulation of ROS and RNS metabolism by NO in peroxisomes through post-translational modifications such as *S*-nitrosylation, nitrosylation, and nitration, as we will discuss later (reviewed in Romero-Puertas and Sandalio, 2016; Sandalio *et al.*, 2019).

### Phytohormones

In the plant cell, there are no specific hormone secretion glands, but there are some naturally produced compounds, in extremely low concentrations, known as phytohormones. These molecules influence every physiological process during plant growth and development, and participate in plant responses to stress and in cell death. As part of the peroxisomal metabolism, the biosynthesis of three plant hormones, namely JA, IAA, and salicylic acid (SA), is regulated in these organelles (Devireddy *et al*., 2021).

The JA family consists of JA, methyl jasmonate (MeJA), the lipid-derived phytohormone jasmonoyl-isoleucine (JA-Ile), and other bioactive oxylipins responsible for regulating plant growth and defence responses to stress and essentially for plant survival in nature (Chini *et al.*, 2018; Zander *et al.*, 2020). JA biosynthesis extends across two cellular compartments, initiating in chloroplasts and completing in peroxisomes. Firstly, the sequential action of different chloroplastic enzymes produces 12-oxo phytodienoic acid (OPDA) and dinor-OPDA (dn-OPDA). Both precursors of JA are transported to peroxisomes facilitated by an ABC transporter. Oxo phytodienoic acid reductase 3 (OPR3) reduces OPDA to 3-oxo-2-(2′-[*Z*]-pentenyl)-cyclopentane-1-octanoic acid (OPC8), and dnOPDA to 3-oxo-2-(2′-pentenyl)-cyclopentane-1-hexanoic acid (OPC6). The resulting compounds are activated to their corresponding CoA esters by OPC:8 CoA ligase l (OPCL1) or the indicated acyl-CoA synthases. The CoA derivatives undergo several β-oxidation cycles (three for OPCS-CoA and two for OPC6-CoA) generating JA-CoA. The cleavage of the CoA fraction finally releases JA (Liu and Timko, 2021). Interestingly, in the absence of OPR3, OPDA enters into the β-oxidation pathway to produce 4,5-didehydro-JA (4,5-ddh-JA) as a direct precursor of JA and JA-Ile (Chini *et al.*, 2018).

Auxins are phytohormones that control mainly plant growth and development, being specifically responsible for cell division and differentiation, fruit development, root establishment, lateral branching, and leaf abscission (Gomes and Scortecci, 2021). Four naturally emerging auxins are present in plants: IAA, IBA, 4-chloroindole-3-acetic acid (4-Cl-IAA), and 2-phenylacetic acid (PAA; Kao *et al*., 2018). IBA is an endogenous auxin with a specific role in lateral root formation and has been suggested to serve as a reservoir of auxin. IBA is metabolized into the bioactive auxin IAA in peroxisomes. The first step includes IBA activation by the addittion of a CoA fraction and then goes through a single β-oxidation cycle resulting in IAA-CoA and an acetyl-CoA. To obtain the active IAA, IAA-CoA is hydrolysed by the activity of a thioesterase. An IBA chemical analogue called 2,4-dichlorophenoxyacetic acid (2,4-D) can be produced from 4-(2,4-dichlorophenoxy) butyric acid (2,4-DB) following the same cascade of enzymatic reactions and differing only in the initial step (Pan *et al.*, 2020). Additionally, IAA signalling can interact with photorespiratory H_2_O_2_-dependent signalling, as reported by Kerchev *et al*. (2015) who observed that supplying exogenous IAA prevented cell death induced by growing Arabidopsis *cat2* mutants under continuous light.

SA is considered an important phytohormone that regulates various physiological aspects in plants including vegetative growth, seed germination, flowering, senescence, environmental stress, and defence responses against pathogens, regulating the activation of local and systemic defence responses against infections (Klessig *et al.*, 2018; Van Butselaar and Van den Ackerveken, 2020). Despite its importance, SA biosynthesis is not well understood. Plants are suggested to possess two pathways to generate SA, named the ICS (isochorismate synthase) and PAL (phenylalanine ammonia-lyase) pathways, and both initiate from chorismic acid produced in chloroplasts. In the ICS pathway (90% of SA biosynthesis), the first step is the conversion of chorismate to isochorismate, by the ICS enzyme. Then, isochorismate is exported to the cytosol and transformed to isochorismate-9-glutamate with the action of enhanced disease susceptibility 5 (EDS5) and AVRPPHB SUSCEPTIBLE 3 (PSB3). The non-enzymatic decomposition of isochorismate-9-glutamate then yields salicylate and 2-hydroxy-acryloyl-*N*-glutamate as final products. On the other hand, the PAL pathway (10% of SA biosynthesis) needs the amino acid phenylalanine as an intermediate compound. This pathway comprises multiple sequential enzymatic steps suggested to occur in peroxisomes, among which are the conversion to *trans*-cinnamic acid and its processing via β-oxidation (Mishra and Baek, 2021).

### Polyamines

PAs, which are low-molecular-weight positively charged biogenic amines, show a high ability to interact with signalling molecules such H_2_O_2_ and NO, and other plant hormones such as ABA, cytokinins, auxins, and ethylene, to regulate plant development and stress responses (Planas-Portell *et al*., 2013; Agudelo-Romero *et al*., 2013; reviewed in Wang *et al*., 2019). The most prominent and well-known PAs in plants are putrescine (Put), spermidine (Spd), and spermine (Spm) (reviewed in Wang *et al*., 2019). Although PA biosynthesis has been thoroughly studied, less information is available on PA catabolism. PAs are oxidatively deaminated by two main amine oxidases, copper-containing amine oxidases (CuAOs) and flavin-containing PA oxidases (PAOs; Planas-Portell *et al*., 2013; Wang *et al*., 2019). A group of CuAOs has been located in peroxisomes from different plant species (Planas-Portell *et al*., 2013; Wang *et al.*, 2019), with two isoforms being located in peroxisomes from Arabidopsis (AtCuAO2 and AtCuAO3; Planas-Portell *et al.,* 2013). Interestingly, most of the peroxisomal PAOs are involved in the back-conversion pathway, producing Spd from Spm and Put from Spd, in addition to 3-aminopropionaldehyde and H_2_O_2_ (Panas-Portell *et al*., 2013; Wang *et al*., 2019).

### Less explored signalling molecules

Different metabolites produced by classical metabolic pathways occurring in peroxisomes, such as photorespiration, have been identified as regulatory signals (reviewed by Timm and Hagemann, 2020). In this way, it has been shown that Ser impairs photorespiratory transcripts (Timm *et al.*, 2013) while low Ser levels may induce the expression of different Ser biosynthesis pathways (Modde *et al.*, 2017). Furthermore, hydroxypyruvate reductase 1 (HPR1) impairment drastically decreases the leaf carbohydrate content and affects sulfur metabolism (Timm *et al.*, 2021). Other new signalling mechanisms are being discovered in plant peroxisomes, such as benzaldehyde biosynthesis, by the β-oxidation pathway, which has been associated with plant pheromone communication, defence responses, and attraction for pollinators (Huang *et al.*, 2022). Furthermore, the degradation of phytol, a compound derived from chlorophyll by α-oxidation, has also been located in peroxisomes (Gutbrod *et al.*, 2021). Additionally, it has been shown that a glycosylase and a kinase catabolize pseudouridine in peroxisomes to prevent the accumulation of toxic pseudouridine monophosphate (Chen and Witte, 2020).

Reactive carbonyl species (RCS) produced by the oxidative degradation of lipid peroxides where ROS, mainly ·OH, are involved, and that can lead to carbonylation of proteins by a covalent addition of a carbonyl group (Mano and Biswas, 2018) have been described in peroxisomes (Mano and Biswas, 2018; Sandalio *et al*., 2023). Although initially RCS and carbonyl groups were associated with oxidative stress markers, new evidence supports that these molecules may be part of the ROS-dependent signalling pathway (reviewed in Biswas and Mano, 2021; Lee and Kim, 2024). It has been recently shown that damaged proteins of PSII in an Arabidopsis line deficient in unfolding activity for PSII repair induce SA signalling (Duan *et al.*, 2019), involving chloroplasts in the initial crosstalk of JA and SA in plant response to stress (Bali *et al.*, 2023). An increase in peroxisomal carbonylated proteins, related to ROS metabolism, the glyoxylate cycle, fatty acid β-oxidation, and cell death, has been described under different stress conditions and senescence, in different plant species (Romero-Puertas *et al.*, 2002; Nguyen and Donaldson, 2005; McCarthy-Suárez *et al.*, 2011; Mano *et al.*, 2014). Although reports on peroxisomal-dependent RCS signalling have not been described so far, the presence in peroxisomes of carbonyls and RCS scavengers, such as GSH and GSTs, which can produce RCS–GSH conjugates (Biswas and Mano, 2021; Sandalio *et al*., 2023); glyoxalase, that detoxifies methylglyoxal (Quan *et al*., 2010), one of the RCS identified in plants (Mano and Biswas, 2018); and aldehyde oxidases (Biswas and Mano, 2021), supports the possible existence of a RCS-dependent signalling pathway in peroxisomes.

Reactive sulfur species (RSS) acts also as signalling molecules in plant and animal systems (Gotor *et al.*, 2019; Lau and Pluth, 2019). H_2_S is one of the best studied RSS and can modify proteins by binding to the metal in metalloproteins and by oxidizing cysteine thiol groups (persulfidation). Although a source for H_2_S has not been identified in peroxisomes, different targets of persulfidation have been described in these organelles. Thus, antioxidant systems such as CAT3 and CSD3, enzymes involved in FA β-oxidation such as ACX1, 3, 4, and 6, and sulfite oxidase have been described as persulfidated (Aroca *et al.*, 2017). In fact, a positive regulation of photorespiratory enzyme activities, such as GOX and CAT, by sulfide has been shown under non-photorespiratory conditions (García-Calderón *et al.*, 2023). These results suggest that persulfidation may regulate ROS metabolism in peroxisomes, thus affecting peroxisomal-dependent signalling. Nevertheless, whether peroxisomes may regulate RSS-dependent signalling needs further research. H_2_S may permeate through the peroxisomal membrane from the cytosol, may be produced non-enzymatically in the organelle (Cao *et al.*, 2019), or an additional source for H_2_S may be located in peroxisomes, with CAT being a possibility, as *in vitro* analyses showed H_2_S production by this enzyme under hypoxic conditions and in the presence of NADPH (Olson *et al.*, 2017).

The peroxisomal reactive species interactome has been recently reported as a multilevel redox regulatory system allowing identification of the families of reactive species in these organelles and the interactions with each other and with their respective targets and antioxidants, offering an overview of peroxisome complexity in terms of metabolic plasticity and signalling (Sandalio *et al*., 2023).

### Post-translational modification of peroxisomal proteins

Post-translational modifications (PTMs) of proteins constitutes a fast and low-cost process to modify protein activity, stability, location, and interactors, therefore regulating metabolic pathways and signalling processes involving these proteins. In particular, ROS- and RNS-dependent PTMs have attracted the attention of the scientific community in the last decades due to the versatility of this modification allowing reversibility of redox changes of Cys residues to act as regulatory switches. Peroxisomal proteins regulated by ROS/RNS-dependent PTMs include proteins involved in the photorespiratory cycle and in the antioxidant system, among others (reviewed in Sandalio *et al*., 2019). However, the functional consequences of these PTMs are not well known, and only a few of them have been investigated. It has been reported that CAT *S*-nitrosylation and nitration decrease its activity in pea leaves (Ortega-Galisteo *et al.*, 2012) and pepper fruits (Chaki *et al*., 2015). On the other hand, CAT has been reported to catalyse peroxynitrite-dependent phenolic nitration, and it has even been proposed that CAT protects *Escherichia coli* from peroxynitrite toxicity (Kono *et al*., 1998). Additionally, protein phosphorylation is also a critical step in many signal transduction pathways and is a reversible process regulated by kinases and phosphatases. The peroxisomal phosphoproteome include antioxidants such as MDHAR 1 and 4, CAT, SOD3, ACX4, ACX6, and NADP-isocitrate dehydrogenase (reviewed in Kataya *et al*., 2019; Sandalio *et al*., 2019). Other protein PTMs such as persulfidation (as previously described), acetylation, glycosylation, or ubiquitination have peroxisomal antioxidants and ROS producers as targets (reviewed in Sandalio *et al*., 2019). For instance, peroxisomal APXs and CATs are targets for ubiquitination (Sharma *et al*., 2016) and acetylation. ACX1 and DHAR are also targets for acetylation and GOX for glycosylation (Sandalio *et al*., 2019), although most of these data require experimental confirmation and further analyses to assess functionality of these modifications.

## Peroxisomal-dependent retrograde signalling under control conditions and in plant stress responses: ROS and RNS function

Upon stress exposure, plants use so-called retrograde signalling (communication between the organelles and the nucleus; Dietz *et al.*, 2016) as well as so-called anterograde signalling (nucleus to organelle communication) to trigger the correct energy use and regulate redox homeostasis (Crawford *et al.*, 2018). Retrograde signals from mitochondria and chloroplast in stress responses are better understood (Wang *et al.*, 2020) than those in peroxisomes. However, the inhibition of CATs has been recently connected with retrograde signals from peroxisomes, denoting the importance of peroxisomal-derived H_2_O_2_ in plant responses to stress and the induction of programmed cell death (Mhamdi *et al.*, 2010a; Terrón-Camero *et al.*, 2022). Although it has been shown that RNS are produced in peroxisomes, signalling dependent on peroxisomal RNS is difficult to analyse due to the complexity and lack of knowledge/specificity of the enzymes involved in their biosynthesis and because the available information is scarce.

### Transcriptomic and metabolomic analyses

Different types of ROS and their specific origin may induce specific transcriptomic responses (Sewelam *et al.*, 2014), and transcriptome analyses of mutant lines associated with a specific organelle have been used to unravel the signalling mechanisms dependent on each organelle (Phua *et al.*, 2021). The higher stability and capability of diffusion of H_2_O_2_ compared with other ROS make this molecule an optimal signalling compound. Thus, the transcriptome of Arabidopsis *cat2* mutants, affected in the main form that metabolizes H_2_O_2_ produced by peroxisomal metabolism, have been extensively analysed under control and different stress conditions as a model for analysing peroxisomal H_2_O_2_-dependent signalling (Table 1; Sandalio *et al*., 2023). Furthermore, changes in the transcriptome in *cat2* mutants showed uniqueness compared with the changes observed by H_2_O_2_ produced in chloroplasts (Sewelam *et al*., 2014) suggesting that there is a specific transcriptomic footprint associated with peroxisomes (Rosenwasser *et al.*, 2013). As one of the main effects after a high increase in CO_2_ is the decrease of photorespiration, with a 4-fold decrease in H_2_O_2_ levels in the cell (Foyer and Noctor, 2020), the shift from high CO_2_ to normal air CO_2_ concentrations has been widely used to analyse peroxisomal H_2_O_2_-dependent signalling (Table 1). The effects of this shift are more obvious in *cat2* mutants, which actually showed a similar phenotype compared with the wild type growing under high CO_2_ conditions, while growth under normal air CO_2_ concentrations induces redox perturbations and activation of oxidative-dependent signalling pathways in the mutants (Queval *et al.*, 2007). High light has also been widely used to study peroxisomal H_2_O_2_-dependent signalling, as it has been shown that CAT-deficient mutants result in decreased H_2_O_2_ scavenging after this stress (Vandenabeele *et al.*, 2003). In fact, CAT-deficient plants from different species or treatment with aminotriazole, a CAT inhibitor (Gechev *et al.*, 2005), showed an altered antioxidant redox state with respect to the non-treated plants, driving a signal transduction pathway and even activating a cell death programme (Noctor *et al.*, 2002; Vandenabeele *et al.*, 2003; Mhamdi *et al.*, 2010a; Foyer and Noctor, 2020). The triple *cat1cat2cat3* mutant displayed severe disturbances in the redox state and showed growth problems under control conditions, with genes involved in plant stress responses differentially regulated (Su *et al.*, 2018). These results supported a significant role for CATs linked to H_2_O_2_-dependent signalling. Therefore, peroxisomal H_2_O_2_-dependent signalling obtained by the different transcriptomic analyses showed regulation of a wide variety of metabolic processes but those involving protein repair in plant stress responses leading to plant acclimation may be highlighted (Mhamdi *et al.*, 2010a; Sandalio and Romero-Puertas, 2015; Su *et al.*, 2019). A meta-analysis carried out with different transcriptomic data available involving mutants or treatments related to peroxisomal H_2_O_2_ metabolism showed a list of common genes categorized in the ‘response to stress-stimulus’ gene Ontology (GO) group, most of them regulated under different abiotic stress situations (Terrón-Camero *et al.*, 2022). Furthermore, different transcription factors rapidly regulated after high CO_2_ to normal air shift have been well established as regulators of plant responses to abiotic stress (Vanderauwera *et al.*, 2005; Mhamdi *et al.*, 2010a; Su *et al.*, 2018). Further characterization of these transcription factors will contribute to understanding how environmental signals and the metabolic state of peroxisomes are translated at the molecular level to the nucleus.

**Table 1.** Metabolomic analyses of mutants affected in peroxisomal ROS metabolism and transcriptomic analyses of different mutants in a *cat2* background

Mutant(s)	Stress	Main results	Reference
*cat2/sid2/cat2sid2 *(*Ath*)	SD/LD	Cell death associated with the *cat2* phenotype is dependent on the daylength and is mediated by the isochorismate pathway of SA synthesis, which is induced in the mutants.	Chaouch *et al*. (2010)*
*cat2/icdh/cat2icdh *(*Ath*)	pathogen	Cytosolic NADP(H) status is needed for the network that activates changes in cellular redox state after pathogen infection and the absence of ICDH exacerbate the phenotype and redox perturbation observed in *cat2 *mutants	Mhamdi *et al*. (2010c)*
*cat2/rbohF/cat2rbohF *(*Ath*)	Shift high CO_2_/normal air	RBOHF is particularly important in coupling intracellular ROS to regulate downstream changes in redox state, SA, and cell death after pathogen attack	Chaouch *et al*. (2012)
*cat2/gox1/gox2/cat2gox1/cat2gox2 *(*Ath*)	High light, shift high CO_2_/normal air	Lack of GOX1, which is the main enzyme metabolizing glycolate, attenuates the cell death phenotype of *cat2* mutants under photorespiratory conditions.	Kerchev *et al*. (2016)*
*hpr1 *(*Ath*)	SD/LD	A severe decrease of leaf carbohydrate status and an impact on sulfur metabolism is observed by a moderate impairment of photorespiration. Lack of HPR1 induces high CO_2_ release	Timm *et al*. (2021)
*AsCAT/AsAPX/AsCATAsAPX* (*Nt*)	Paraquat/pathogen	The lack of CAT and cytosolic APX1 induced co-ordinately metabolic and defence genes while it suppressed photosynthetic activity	Rizhsky *et al*. (2002)
*cat2/gr1/cat2gr1* (*Ath*)	Shift high CO_2_/normal air SD/LD	Lesion formation, accumulation of SA, induction of PR genes, and JA-dependent signalling in *cat2* mutants are dependent on the redox status of glutathione	Mhamdi *et al*. (2010b)
*cat2/apx1/cat2apx1* (*Ath*)	High light	CAT2 and cytosolic APX1 play a key role in protecting the plant genome against photorespiratory-dependent H_2_O_2_-induced DNA damage	Vanderauwera *et al*. (2011)
*cat2/shr-6/cat2shr-6* (*Ath*)	High light, shift high CO_2_/normal air	Photorespiratory H_2_O_2_ production is limited by SHR deficiency impacting the cellular redox homeostasis and integrating stress responses and development	Waszczak *et al*., (2016)

Metabolomic analyses (grey) in mutants affected in peroxisomal ROS metabolism (most of them are accompanied by transcriptomic analyses: highlighted by an *) and transcriptomic analyses (white) of different mutants in a *cat2* background

For transcriptomic analyses with single mutants affected in peroxisomal ROS metabolism, see Sandalio *et al*. (2023). Chaouch *et al*. (2010) and Kerchev *et al*. (2016) also reported transcriptomics analyses. *Ath*, *Arabidopsis thaliana*; *Nt*, *Nicotiana tabacum*; *cat*, *catalase*; *gox*, *glycolate oxidase*; *hpr*, *hydroxy pyruvate reductase*; *apx*, *ascorbate peroxidase*; *shr*, *short root*.

Interestingly, signalling activated in Arabidopsis and tobacco lines deficient in CAT is also related to plant responses to biotic stress as it has been described by metabolomic and transcriptomic analyses (Chaouch *et al.*, 2010, 2012; Su *et al.*, 2019; Table 1). In fact, *cat2* mutants showed enhanced resistance to bacteria through SA and camalexin accumulation, triggering lesions that mimic hypersensitive responses, which are daylength dependent (Chaouch *et al.*, 2010). Recently, it has been shown that SA, in turn, inhibits CAT2 to promote an increase in H_2_O_2_ levels after pathogen attack, which repressed the levels of the auxin precursor tryptophan. Furthermore, CAT2 promoted acyl-CoA oxidases ACX2 and ACX3 to increase JA levels upon pathogen infection. Thus, *cat2* mutants showed an increased biotroph resistance but a decreased resistance to necrotrophs (Yuan *et al.*, 2017). Peroxisomal H_2_O_2_ and auxin homeostasis have also been linked through the action of an IBA UDP-glucosyltransferase (UGT74E2; Tognetti *et al.*, 2010).

Transcriptomes of double mutants involving *cat2*, which allowed for increased internal H_2_O_2_ levels and a perturbed redox state in the other mutant background, have also been analysed. For example, *cat2 gr1* is also affected in GR activity, which is not only involved in maintaining the reduced state of glutathione, a main cellular thiol, but also involved in removing H_2_O_2_ through the ASC–GSH cycle. Thus, it has been shown that the regulation of genes involved in SA and JA signalling were dependent on the glutathione redox status and the expression of different glutaredoxins and GSTs, which were modified in the double mutant (Mhamdi *et al.*, 2010b). Interestingly, double mutants deficient in peroxisomal CAT and cytosolic APX1 were more resistant to oxidative stress than single mutants (Rizhsky *et al.*, 2002; Vanderauwera *et al.*, 2011). Transcriptomic analysis showed that in the double mutants a DNA damage response was activated, suggesting that both CAT2 and APX1 are necessary and have a major role in protecting DNA from damage from photorespiratory H_2_O_2_ (Vanderauwera *et al.*, 2011). On the other hand, metabolomic analysis of the double mutant *cat2 icdh* underscored a role for NADP-dependent isocitrate dehydrogenase (cICDH) in plant responses to pathogens linked to redox signalling (Mhamdi *et al.*, 2010c). Further metabolomic analysis on double mutants involving CAT and the main sources of ROS production after pathogen attack, RBOHD and RBOHF, showed that RBOHF regulated the metabolomic response to bacterial pathogens and plant resistance (Chaouch *et al.*, 2012), in addition to the already known function in programmed cell death. Mutants affected in the peroxisomal photorespiratory glycolate oxidase1 (GOX1) but not GOX2, attenuated the phenotype of *cat2* lines, suggesting that GOX1 produces more H_2_O_2_ under photorespiratory conditions and its absence can alleviate damage in *cat2* mutants (Kerchev *et al.*, 2016). Further transcriptomic and metabolomic analyses showed the inability of *gox1* and *gox2* to process photorespiratory glycolate in a *cat2* background, with *gox1* more affected with a more highly impaired H_2_O_2_ production and a more severe regulation of redox homeostasis (Kerchev *et al.*, 2016). In addition, transcriptomic analysis on *shr-6* mutants affected in SHORT-ROOT (SHR) protein and double *cat2 shr-6* showed that SHR is involved in H_2_O_2_-dependent gene expression, ascorbate decrease, and GSH oxidation in the *cat2* background under photorespiratory conditions, integrating stress responses and development (Waszczak *et al.*, 2016).

Transcriptomes and/or metabolomes of mutants affected in peroxisomal proteins related to other signalling molecules and/or phytohormones are less known probably due to the difficulty in identifying a specific peroxisomal imprint (Table 1). Peroxisomal ACX1-dependent H_2_O_2_ regulated IAA metabolism, the E3-RING ubiquitin ligase, and the proteasome complex, which was involved in plant responses to the herbicide 2,4-D (Romero-Puertas *et al.*, 2022). Furthermore, *PEX11A* RNAi Arabidopsis mutants, that are not able to form peroxules, had the regulation of genes related to ROS metabolism disrupted, in plant responses to stress, suggesting that PEX11A might participate in peroxisomal-dependent signalling (Rodríguez-Serrano *et al.*, 2016).

### Proteomic analyses

As peroxisomes lack DNA, peroxisomal proteins should be encoded in the nucleus and imported into the organelle mainly through the peroxin (PEX) proteins. After peroxisomal matrix proteins are synthesized in the cytosol, the proper importation into the matrix of peroxisomes is in general, facilitated by their peroxisomal targeting signals (PTSs) that most of them possess. PTSs are recognition sequences at the C-terminus (the most common tripeptide PTS1, with an SKL sequence) or at the N-terminus (a nonapeptide PTS2 present in a smaller number of proteins) of the respective cargo proteins (Sparkes and Baker, 2002; Kaur and Hu, 2011; Reumann *et al.*, 2016). Experimental proteomics carried out in the last years have allowed the identification of peroxisomal proteins made up of ~200 proteins and have added new functions to this organelle (reviewed in Quan *et al.*, 2010, 2013; Kaur and Hu, 2011; Reumann, 2011). Plant peroxisome proteome changes depend on the developmental stage of the plant, with different metabolic cycles being predominant at each stage, such as the glyoxylate cycle in seedlings and photorespiration in green leaves (Pan *et al.*, 2020). Furthermore, different analyses of the peroxisomal proteome showed a high number of proteins targeted by different PTMs, which would explain the ability of peroxisomes to rapidly change their metabolism and respond to stress (reviewed in Sandalio *et al.*, 2019). We do not discuss here proteome analyses related to functions and pathways associated with the peroxisomal proteins, as several excellent reviews are available (Reumann and Bartel, 2016; Kao *et al.*, 2018; Pan and Hu, 2018), but rather proteomic studies on peroxisomal proteins linked to signalling.

One of the most studied peroxisomal proteins is CAT, which regulates peroxisomal H_2_O_2_ levels and whose interactome is very complex and, in part, unexpected. Within the CAT interactome there are proteins that are located outside peroxisomes and there are other proteins that act as chaperones for CAT (see review by Baker *et al.*, 2023). The link between CAT and photorespiratory-dependent H_2_O_2_ signalling is the SA-regulated GOX–CAT interaction shown in rice leaves (Zhang *et al.*, 2016). Apparently, this association was also essential in the control of stomatal aperture (Yamauchi *et al.*, 2019). A protection of CAT2 has been shown under abiotic stresses by a peroxisomal heat shock protein (Hsp17.6CII; Li *et al.*, 2017) and protection between CAT and isocitrate lyase has also been reported during seed germination in castor bean glyoxysomes (Yanik and Donaldson, 2005). NO CATALASE ACTIVITY 1 (NCA1) also acted as a chaperone and is needed for proper CAT folding and activation either before or after import into peroxisomes, being essential in plant responses to biotic and abiotic stresses (Hackenberg *et al.*, 2013; Li *et al.*, 2015). Additional research suggested that NCA1 and Hsp17.6CII chaperones work additively in the cytosol and peroxisomes, respectively (Li *et al.*, 2017). Arabidopsis SOS2 (salt overly sensitive 2) and LAP2 (leucine aminopeptidase 2) also interact with CAT proteins under salt stress, although the mechanisms and functionality are not clear (Verslues *et al.*, 2007; Zhang *et al.*, 2021). Recently, the interaction of the tomato methionine sulfoxide reductase SlMsrB2 with CAT2 has been shown to be involved in drought tolerance through modulation of ROS accumulation. Interaction with other proteins such as LSD1 (lesion simulating disease 1; Li *et al.*, 2013), CDPK8 (cytosolic calcium-dependent kinase; Zou *et al.*, 2015), STRK1 (salt tolerance receptor-like cytoplasmic kinase 1; Zhou *et al.*, 2018), OsCPK10 (plasma membrane-associated calcium-dependent kinase; Bundó and Coca, 2017), and NRX1 (Nucleoredoxin 1; Kneeshaw *et al.*, 2017) promoted CAT activity directly or indirectly, suggesting that these interactions may induce CAT stability and/or affect the CAT PTM pattern (Baker *et al.*, 2023), underscoring the complexity of the specific regulation system for CAT. Furthermore, several CAT-interacting proteins, including plant and pathogen effectors (Baker *et al*., 2023), are located in the cytosol or nucleus, suggesting that, especially under stress conditions, CAT could be retained in the cytosol (Foyer *et al.*, 2020) or could be released from peroxisomes at some point (Fujiki *et al.*, 2017). In fact, in mammalian cells, oxidative stress induced PEX14 phosphorylation avoiding CAT interaction with the importomer complex and allocation of CAT into peroxisomes (Okumoto *et al.*, 2020). Recently, it has been shown that CAT2 from Arabidopsis could be located in the nucleus due to an alteration of the C-terminus of the protein that could affect tetramerization (Al-Hajaya *et al.*, 2022). In fact, several pathogen effectors may interact with CAT, not only regulating plant H_2_O_2_ degradation but also reallocating CAT in the nucleus (Mathioudakis *et al.*, 2013; Zhang *et al.*, 2015; Murota *et al.*, 2017; Gao *et al.*, 2021; Yuan *et al.*, 2021), whose functional significance is not well understood.

## Peroxisomal plasticity and signalling

Plant peroxisomes have a high plasticity, being able to change their size, morphology, number, and even speed of movement depending on metabolic and environmental signals. The regulation of these changes, their functionality, and the advantages of this plasticity are not well known, however. It is well established that peroxisomes move along actin filaments requiring myosin motor proteins (Perico and Sparkes, 2018) and actin-binding proteins such as PEROXISOME AND MITOCHONDRIAL DIVISION FACTOR1 (PMD1), which are involved in peroxisomal division and the cellular distribution of peroxisomes in response to environmental changes. In plants, the motility and morphology of peroxisomes have also been correlated with the motility of the endoplasmic reticulum (ER; Barton *et al.*, 2013).

**Fig. 1.**
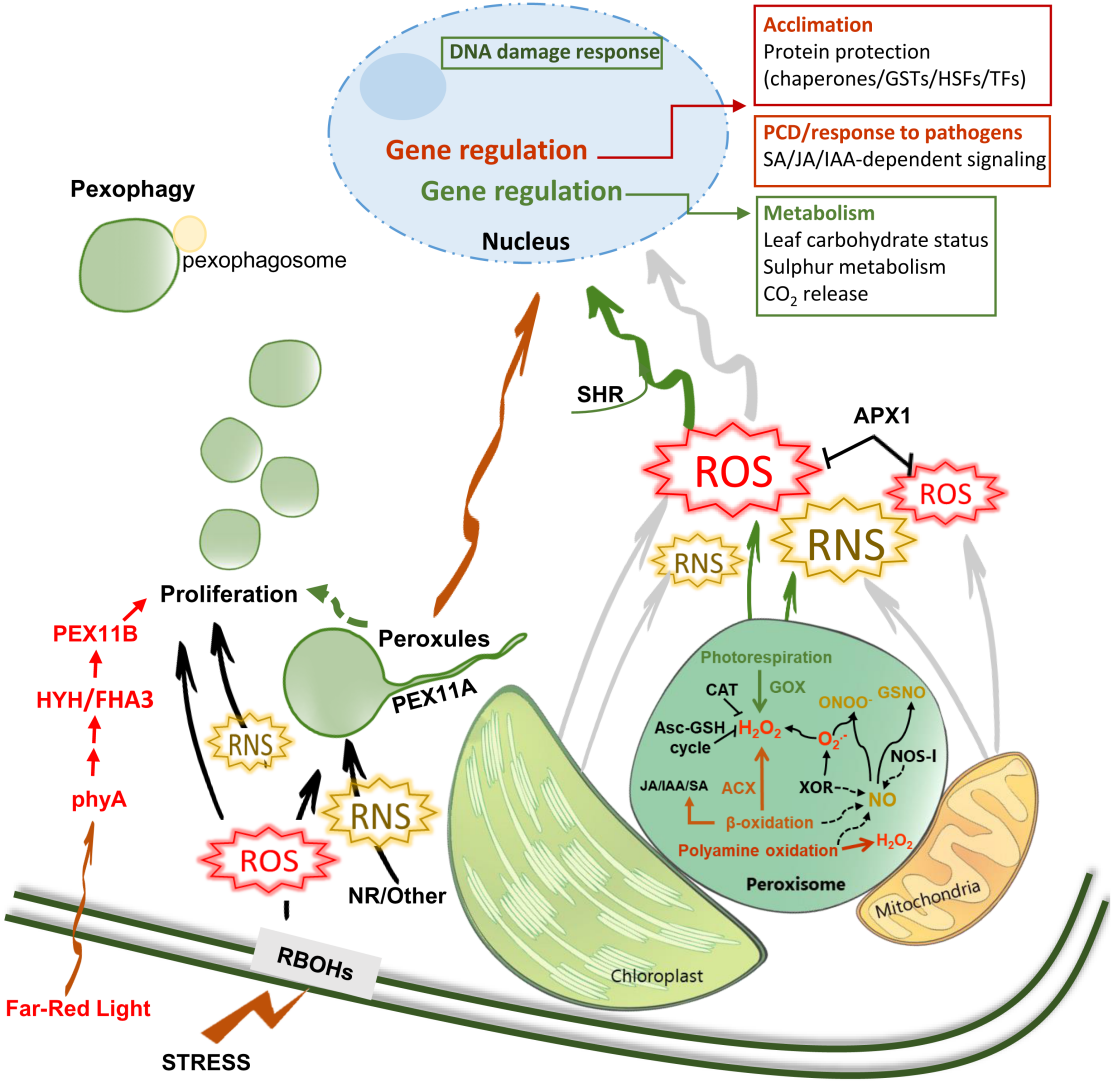
Scheme showing peroxisomal-dependent signalling processes under normal and stress conditions. Peroxisomes collaborate and communicate with other organelles or cell locations, such as mitochondria, chloroplasts, and the plasma membrane, to organize plant metabolism under normal conditions and in plant responses to stress. Under normal conditions, peroxisomes regulate leaf carbohydrate status, sulfur metabolism, and CO_2_ release (in green). In the absence of CAT, mechanisms related to programmed cell death (PCD) and hormonal-dependent responses to pathogens are activated (in brown). In addition, stress conditions promote the generation of ROS, including hydrogen peroxide (H_2_O_2_) and superoxide (O_2_·^−^), and RNS, including nitric oxide (NO), nitrosoglutathione (GSNO), and peroxynitrite (ONOO^−^). Peroxisomal-dependent ROS activate protein protection mechanisms under stress (in brown). ROS and RNS also activate the peroxin PEX11A, which promotes the formation of peroxules, that in turn could control ROS/RNS accumulation and ROS/RNS-dependent gene expression. The proliferation of peroxisomes is also induced under different stress situations involving ROS/RNS generation, in particular far-red light-induced peroxisome proliferation by a signalling network (in red) controlled by phytochrome A (phyA), the transcription factor HYH, and PEX11b. Furthermore, the pexophagy process may be activated under stress situations to regulate peroxisomal homeostasis and quality, and possibly ROS/RNS accumulation in the cell. Dashed lines show putative NO sources in peroxisomes. APX1, ascorbate peroxidase; CAT, catalase; GOX, glycolate oxidase; IAA, indole-3-acetic acid; JA, jasmonic acid; NOS-l, nitric oxide synthase-like; SA, salicylic acid; SHR, short root; XOR, xanthine oxidoreductase.

In Arabidopsis, peroxisome proliferation is a common feature against different stress conditions particularly associated with ROS accumulation (Fig. 1), such as salinity, heavy metals, UV light, ozone, drought, clofibrate, ABA, senescence, or pathogen infections (for reviews, see Kao *et al.*, 2018; Sandalio *et al.*, 2021). Peroxisome proliferation is regulated by peroxisomal membrane proteins termed peroxins PEX11 (A–E), which drive the elongation of peroxisomes, followed by the action of dynamin-related proteins (DRPs; DRP3A, DRP3B, and DRP5B) and fission proteins (FIS1), which produce constriction and finally the fission of the peroxisomes, giving rise to new organelles (Kao *et al.*, 2018). Dynamic changes in the PEX11 family have been reported depending on the stress factor: salinity up-regulated *PEX11A* and *PEX11C* in *A. thaliana* (Fahy *et al.*, 2017); *PEX11A* and *PEX11E* were up-regulated in response to Cd exposure (Rodríguez-Serrano *et al.*, 2016; Terrón-Camero *et al.*, 2020); *PEX11B*, *PEX11C*, and *PEX11D* were up-regulated by hypoxia (Li and Hu, 2015), and *PEX11B* was induced by high light (Desai and Hu, 2008). However, the transcriptional regulators of these genes and peroxisome proliferation have not been elucidated, except for the HYH/FHA3 transcription factor which regulates *PEX11B* under photomorphogenesis requiring phytochrome A (phyA) (Fig. 1; Desai and Hu, 2008). Frick and Strader (2018a, b) demonstrated that MAP KINASE17 (MPK17) is a potential regulator of peroxisome division requiring PMD1, a mechanism independent of DRPs/FIS (Aung and Hu, 2011), in responses to salinity, but not in general stress-dependent peroxisome proliferation. In addition to ROS, in Arabidopsis *nia1nia2*, which showed lower NO levels, peroxisomal proliferation was highly compromised in response to Cd. NO-related Arabidopsis mutants also displayed disturbances in peroxisomal ROS metabolism affecting the number of organelles and peroxisomal-dependent signalling (Terrón-Camero *et al.*, 2020). These phenotypes were associated with changes in the pattern of ROS and NO-dependent PTMs of CAT, suggesting that a fine-tuned balance between ROS and NO is necessary to regulate peroxisome proliferation (Terrón-Camero *et al.*, 2020). Application of γ-aminobutyric acid (GABA) resulted in peroxisome proliferation and transcriptional up-regulation of *PEX11C* and *BETAINE ALANINE DEHYDROGENASE* (*BADH*), which participate in the catabolism of PAs (Şahin *et al.*, 2024). These results suggested that GABA alters ROS homeostasis promoting peroxisome proliferation (Şahin *et al.*, 2024), probably by enhancing H_2_O_2_ and NO production during PA catabolism (for reviews, see Wang *et al.*, 2019; Sandalio *et al.*, 2021). Some benefits from peroxisome proliferation have been suggested: overexpression of OsPEX11 in rice enhanced the tolerance to salinity, increasing the activities of antioxidant enzymes (SOD, POD, and CAT) and proline accumulation, and decreasing the level of lipid peroxidation and the Na/K ratio (Cui *et al.*, 2016). Salt susceptibility also correlated with a decrease of peroxisome proliferation in the *fry1-6* line, lacking inositol polyphosphate 1-phosphatase, an essential component of the stress signalling network (Fahy *et al.*, 2017). Interestingly, Oikawa *et al.* (2022) have reported that phosphatidylinositol 3-phosphate is required to bind the autophagy marker ATG8a to peroxisomes and autophagosomal structures near the peroxisome, thus promoting pexophagy. Therefore, this information contrasts with the decrease of peroxisomal proliferation in *fry1-6* lines (Fahy *et al.*, 2017). The role of protein phosphorylation/dephosphorylation processes in peroxisomal proliferation is an interesting issue that deserves a more in-depth study. However, the increase of peroxisome number by overexpressing peroxisome division factors fails to increase abiotic stress tolerance in Arabidopsis (Mitsuya *et al.*, 2010) and the proliferation of peroxisomes in quinoa plants exposed to heat, drought, or both negatively affected plant yield (Hinojosa *et al*., 2019).

Time-lapse analysis of Arabidopsis plants in responses to exogenous H_2_O_2_, Cd, and As treatment has shown that peroxisomes experience fast (in just a few minutes) changes in their morphology, with the formation of dynamic extensions termed peroxules, which are in contact with chloroplasts and mitochondria (Sinclair *et al.*, 2009; Sandalio *et al.*, 2013; Jaipargas *et al.*, 2016; Rodríguez-Serrano *et al.*, 2016). Following peroxule formation, peroxisomes may elongate, acquire constrictions, and finally divide into several daughter peroxisomes. However, this sequence could not necessarily be applied to all types of stress factors—NaCl and pathogens apparently do not produce peroxules (Frick and Strader, 2018b). According to Zhang *et al.* (2023), peroxisomal elongation is essential for ROS depletion and infectious growth in the rice blast *Magnaporthe oryzae* and is regulated by host-derived ROS production, probably associated with NADPH oxidases. Rodríguez-Serrano *et al*. (2016) demonstrated that Cd treatment induces peroxules which are regulated by RBOH-dependent H_2_O_2_ and PEX11A, and that these extensions are in contact with chloroplasts. Jaipargas *et al.* (2016) also observed the formation of peroxules in contact with mitochondria after a short exposure to high light irradiation, and this process was in parallel to an increase in cytosolic H_2_O_2_. In the leaves of *atg7*(p4) Arabidopsis mutants, peroxules were observed, suggesting that leaves accumulated a high level of ROS (Oikawa *et al*, 2022). Although, so far, peroxules have been observed in contact with chloroplasts and mitochondria, we cannot rule out a connection with other cell compartments.

The function of peroxules is elusive but could contribute to signal transduction by transferring H_2_O_2_ directly to the nucleus, as has been demonstrated in chloroplasts where stromules (dynamic structures similar to peroxules) can transfer H_2_O_2_ from chloroplasts to nuclei as a part of a retrograde signalling process (Caplan *et al.*, 2008, 2015). However, no direct evidence of a peroxule–nucleus contact has been imaged so far. In *Nicotiana benthamiana*, Caplan *et al*. (2015) observed that stromules were induced in the innate immunity responses and connected with the nucleus. The authors demonstrated that stromule formation was dependent on SA and H_2_O_2_, and participated in the transfer of H_2_O_2_ and the N Receptor Interacting Protein 1 (NRIP1) from the chloroplasts to nuclei. Interestingly, *PEX11A* RNAi Arabidopsis mutants, which are unable to produce peroxules, showed disturbances in ROS-dependent signalling with changes in the expression of *GST*, *CAT2*, *CuZn-SOD3*, and the redox-responsive transcription factor1 gene (*RRTF1*), well known to be regulated by ROS. This took place in parallel to changes in malondialdehyde content during the fast responses to cadmium exposure (Rodríguez-Serrano *et al*., 2016). Therefore, peroxules could participate in transducing early ROS-dependent signalling against external stimuli, although the mechanisms involved have not been established. Peroxules could also favour exchange of metabolites or proteins with chloroplasts and mitochondria, which share metabolic pathways with peroxisomes. Therefore, peroxules transfer the sugar-dependent 1 (SDP1) lipase to the lipid body in cotyledons (Thazar-Poulot *et al.*, 2015).

Cells are required to regulate an excessive number of peroxisomes to maintain cellular redox homeostasis. As mentioned before, peroxisome proliferation is a common feature of different abiotic stresses and, as a consequence of that, an excessive accumulation of ROS could take place. An excess of ROS may affect peroxisomal protein oxidation, such as of CAT, therefore promoting oxidative damages in the cell (Romero-Puertas *et al.*, 2002; Calero-Muñoz *et al.*, 2019; Oikawa *et al.*, 2022). Some evidence showed that ROS and oxidative damage to peroxisomes regulate the degradation of oxidized whole peroxisomes by selective autophagy termed pexophagy (Shibata *et al.*, 2013; Yoshimoto *et al.*, 2014; Calero-Muñoz *et al.*, 2019; Oikawa *et al.*, 2022). Therefore, an efficient system of peroxisome quality regulation is an important part of the cellular mechanism to cope with stress conditions and would directly participate in the complex redox regulation of transcriptional responses under stress and development conditions. Recently, Martinek *et al.* (2023) reported that peroxisomes interact with the actin-related protein 2/3 (ARP2/3), which could form a scaffold facilitating the degradation of peroxisomes by autophagy with the involvement of ATG8F. The ubiquitination of proteins such as PEX5, PEX3, and PMP34 was also suggested to activate pexophagy (Akhter *et al.*, 2023). It remains unclear whether ubiquitination plays a signalling role in pexophagy. Knowledge of proteins specifically involved in plant pexophagy is scarce. The chaperone activity of the peroxisomal protease LON2 was demonstrated to inhibit pexophagy (Farmer *et al*., 2013; Young and Bartel, 2016) but receptors linking peroxisomes to the autophagy machinery remain elusive. Although several pexophagy receptors have been identified in yeast, these proteins lack apparent homologues in plants (Olmedilla and Sandalio, 2019). According to Oikawa *et al.* (2022), oxidized lipids in the membrane of peroxisomes could be the signal to recognize the oxidized peroxisomes for pexophagy, and they observed that phosphoinositide 3-kinase is involved in pexophagosome formation (Oikawa *et al*., 2022). As reported by Oikawa *et al*. (2022), phosphatidylinositol 3-phosphate would be generated on the peroxisome membrane or would be associated with pre-autophagosomal structures via the action of an undefined factor in response to ROS and it will be recognized by ATG18a, which finally will trigger the formation of pexophagosomes (Oikawa *et al*., 2022).

## Conclusion

In recent years, considerable progress has been made in our knowledge about the cellular processes in which peroxisomes participate. In addition, much data from -omic and cellular techniques have been accumulated involving peroxisomal-dependent signalling and dynamics in the regulation of plant metabolism and in plant acclimation to stress (Fig. 1). Most of what we currently know about peroxisome-dependent signalling is related to H_2_O_2_ due to the specificity of ROS sources and antioxidants in peroxisomes, such as GOX, ACX, and CAT. However, further research into the influence of peroxisomal ROS on oxidative modifications of specific targets and RCS in responses to different stresses and their possible roles regarding damage, protection, and signalling is needed to provide a relevant understanding of the role of peroxisomes in the network of plant development and stress responses. These analyses will allow us to reveal novel pathways and may uncover the mechanisms through which peroxisomes perceive stress and translate these signals to the nucleus. Much more remains to be done, however, related to peroxisomal RNS-dependent signalling due to the complexity and/or lack of knowledge of their specific biosynthesis/metabolic processes. Similarly, signalling dependent on peroxisomal phytohormones and other molecules is practically unexplored. Deciphering the full process involving the peroxisomal perception of the stress, changes in the dynamics and metabolism of the organelles, and activation of plant responses to changes in their environment will deliver basic knowledge for the enhancement of crop yield, quality, and acclimation.
